# RAD‐tag and mitochondrial DNA sequencing reveal the genetic structure of a widespread and regionally imperiled freshwater mussel, *Obovaria olivaria* (Bivalvia: Unionidae)

**DOI:** 10.1002/ece3.8560

**Published:** 2022-01-26

**Authors:** Jamie R. Bucholz, Nicholas M. Sard, Nichelle M. VanTassel, Jeffrey D. Lozier, Todd J. Morris, Annie Paquet, David T. Zanatta

**Affiliations:** ^1^ Biology Department Institute for Great Lakes Research Central Michigan University Mount Pleasant Michigan USA; ^2^ Department of Biological Sciences The University of Alabama Tuscaloosa Alabama USA; ^3^ Biological Sciences Department State University of New York‐Oswego G83A Shineman Center Oswego New York USA; ^4^ Fisheries and Oceans Canada Burlington Ontario Canada; ^5^ Direction de l’expertise sur la faune aquatique Ministère des Forêts, de la Faune et des Parcs Québec Quebec Canada

**Keywords:** freshwater mussels, genetic structure, Laurentian Great Lakes, population genetics, postglacial colonization, RAD‐seq

## Abstract

*Obovaria olivaria* is a species of freshwater mussel native to the Mississippi River and Laurentian Great Lakes‐St. Lawrence River drainages of North America. This mussel has experienced population declines across large parts of its distribution and is imperiled in many jurisdictions. *Obovaria olivaria* uses the similarly imperiled *Acipenser fulvescens* (Lake Sturgeon) as a host for its glochidia. We employed mitochondrial DNA sequencing and restriction site‐associated DNA sequencing (RAD‐seq) to assess patterns of genetic diversity and population structure of *O*. *olivaria* from 19 collection locations including the St. Lawrence River drainage, the Great Lakes drainage, the Upper Mississippi River drainage, the Ohioan River drainage, and the Mississippi Embayment. Heterozygosity was highest in Upper Mississippi and Great Lakes populations, followed by a reduction in diversity and relative effective population size in the St. Lawrence populations. Pairwise *F*_ST_ ranged from 0.00 to 0.20, and analyses of genetic structure revealed two major ancestral populations, one including all St. Lawrence River/Ottawa River sites and the other including remaining sites; however, significant admixture and isolation by river distance across the range were evident. The genetic diversity and structure of *O*. *olivaria* is consistent with the existing literature on *Acipenser fulvescens* and suggests that, although northern and southern *O*. *olivaria* populations are genetically distinct, genetic structure in *O*. *olivaria* is largely clinal rather than discrete across its range. Conservation and restoration efforts of *O*. *olivaria* should prioritize the maintenance and restoration of locations where *O*. *olivaria* remain, especially in northern rivers, and to ensure connectivity that will facilitate dispersal of *Acipenser fulvescens* and movement of encysted glochidia.

## Graphical abstract

Using RADseq and mitochondrial sequencing, we investigated the genetic diversity and structure of an imperiled freshwater mussel species, *Obovaria olivaria*, that uses a similarly imperiled host fish, *Acipenser fulvescens*. We show that *O. olivaria* is partially subdivided into northern and southern groups by the Great Lakes, but that overall structure is largely continuous and driven by geographic (river) distance between populations.

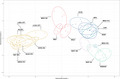

## 1 INTRODUCTION

The conservation of freshwater mussels is essential to the health of freshwater aquatic ecosystem given their important ecosystem services, including biofiltration, nutrient cycling, and sediment formation (Elderkin et al., 2007; Vaughn et al., 2008). Freshwater mussels (Bivalvia: Unionidae) are one of the most imperiled groups of freshwater organisms (Ricciardi & Rasmussen, 1999), and like many species, they face tremendous declines due to global climate change and anthropogenic threats. Pollution, legacy contaminants, and invasive species have led to a dramatic loss in aquatic biodiversity (Haag & Williams, 2014). For freshwater mussels, the pearl and button industry heavily exploited populations in the early 1900s. Subsequent construction of dams led to the destruction of irreplaceable habitat and impeded dispersal of host fish for freshwater mussels, while increased agricultural land use and associated run‐off into aquatic systems has also been detrimental (Haag, 2012). Lastly, in the early 1990s, *Dreissena polymorpha* and *Dreissena rostriformis bugensis* (Zebra and Quagga mussels) became established in the Great Lakes and can out‐compete native freshwater mussels and even form large aggregates that suffocate native mussel species (Lucy et al., 2013). Current management guidelines for imperiled or endangered species strive to preserve unique or rare genetic variation (Fraser, 2008; Jones et al., 2006) and examining trends in the diversity and structure of imperiled freshwater species is crucial to management and recovery planning (FMCS, 2016; Petit et al., 1998).

*Obovaria olivaria* (common name: Hickorynut, Rafinesque, 1820) is a member of the freshwater bivalve family Unionidae and is widely distributed in central North America (COSEWIC, 2011). *Obovaria olivaria* is a species typically found in large rivers from the Mississippi River drainage system and the Great Lakes‐St. Lawrence basin, extending south to Missouri, Arkansas, and Louisiana, east to Quebec, New York, and Pennsylvania, and west to Kansas (Parmalee & Bogan, 1998). While considered least concern by the IUCN, *O*. *olivaria* is considered imperiled (e.g., endangered, threatened, or special concern) across much of its distribution, especially in the Great Lakes region (COSEWIC, 2011; Natureserve, 2021), and is endangered in Canada due to declines in habitat, host fish declines, and the introduction of dreissenid mussels (COSEWIC, 2011).

Like most North American freshwater mussels, *O*. *olivaria* is dioecious (COSEWIC, 2011; Hoeh et al., 1995). A female mussel typically broods glochidia (a parasitic larvae) until a suitable host fish is near before discharging mature glochidia to successfully parasitize the host (Barnhart et al., 2008). Glochidia eventually metamorphose into juveniles and dislodge from the gills of the host fish to drop to the bottom of the riverbed (Oesch, 1995). Once mussels develop into free‐living filter‐feeding adults, their natural movement is severely limited and thus are virtually dependent on the brief period of attachment to the host fish or the supplemental stocking of adult mussels for long‐distance dispersal (Haag & Warren, 2011). Transformation of *O*. *olivaria* glochidia has been documented on *Acipenser fulvescens* (common name: Lake Sturgeon, Rafinesque 1817), and *O*. *olivaria* in the Great Lakes and the St. Lawrence drainage are only known from areas where *A*. *fulvescens* are present (Brady et al., 2004; COSEWIC, 2011). *Obovaria olivaria* may also utilize *Scaphirhynchus platorynchus* (Shovelnose Sturgeon) in parts of its range (Coker et al., 1921), particularly in the Upper Mississippi River basin where *A*. *fulvescens* is rare and considered vulnerable (Knights et al., 2002; Natureserve, 2021) and *S*. *platorynchus* is more abundant and secure (Knights et al., 2002; Natureserve, 2021).

Understanding how genetic diversity is structured among *O*. *olivaria* populations across this large distribution will be an important component of future conservation and management plans (Fraser, 2008; Jones et al., 2006). As discussed above, the distribution of freshwater mussels is tied to the distribution and movement of their host fish species (Barnhart et al., 2008; Leibold et al., 2004; Newton et al., 2008; Schwalb et al., 2013; Zanatta & Murphy, 2006; Zanatta & Wilson, 2011). Sturgeons are capable of dispersing over 200 km during spawning periods (Auer, 1996; Wildhaber et al., 2011). Thus, the large distribution of *O*. *olivaria* may be driven by long‐distance dispersal of its glochidia by sturgeon hosts, combined with stream capture events of waterways during the Pleistocene glaciation (Brady et al., 2004; Coker et al., 1921; Underhill, 1986). These factors may also contribute to a history of extensive gene flow within *O*. *olivaria*. However, given limited contemporary connectivity among drainages and the potential for increasingly limited dispersal of fish hosts discussed above, distinguishing whether populations exhibit substantial connectivity (e.g., isolation‐by‐distance) or more discrete genetic structure among rivers will be important. Beyond impacting gene flow and population genetic structure, recent and historical changes in connectivity among river drainages can leave signatures on levels of genetic diversity, and quantifying variation in diversity among less threatened populations (e.g., Mississippi River) and endangered populations (e.g., St. Lawrence River) could provide important information on how genetic diversity relates to conservation status.

The objectives of this study are to (1) examine the genetic diversity of *O*. *olivaria* across its range and identify populations or regions harboring relatively low genetic diversity and (2) examine the genetic structure of *O*. *olivaria* across its range to identify patterns of connectivity or presence of discrete population structure and dispersal barriers that can assist in focal management planning. We employ two complementary molecular approaches, mitochondrial DNA (mtDNA) sequencing and restriction site‐associated DNA sequencing (RAD‐seq), to examine the phylogeography, genetic structure, and genetic diversity of *O*. *olivaria* populations throughout its range. Mitochondrial DNA has been widely used in range‐wide phylogeographic studies for decades (Avise et al., 1987; Beebee & Roe, 2008), including numerous analyses of freshwater mussels to determine population structure at broad geographic scales (Inoue & Berg, 2017; Inoue et al., 2014; Zanatta & Harris, 2013), but represents only a single genetic marker. RAD‐seq is a powerful high‐throughput sequencing approach that is increasingly used in population genetics analyses (Andrews et al., 2016). By providing large numbers of genome‐wide single‐nucleotide polymorphisms (SNPs), RAD‐seq is of great value in population and conservation genetics because of the improved power to detect differentiation even in small or geographically restricted populations without requiring prior genomic resources (Andrews et al., 2016; Kang et al., 2017). Considering the imperiled status and rarity of *O*. *olivaria* in many parts of its distribution, RAD‐seq offers a robust and innovative means to detect genomic differences among populations. Assessing both mitochondrial and genomic diversity in *O*. *olivaria* should provide insights into the population structure and diversity that will be valuable for management of this species.

## 2 MATERIALS AND METHODS

### 2.1 Sampling locations

**FIGURE 1:**
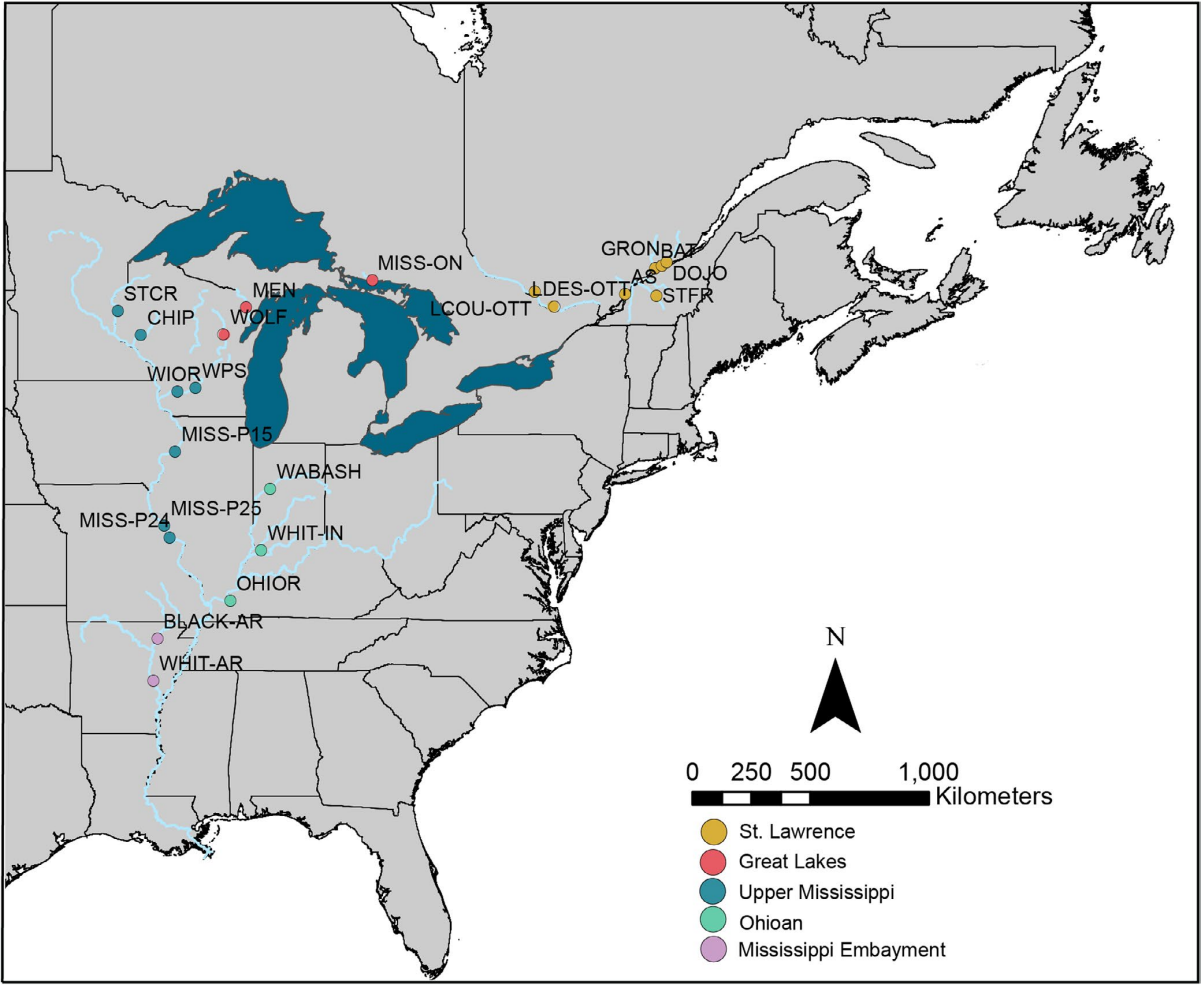
Distribution of *Obovaria olivaria* collection sites color‐coded by drainage. Collection site codes as in Table 1

*Obovaria olivaria* samples were collected from 19 sites across its distribution in the upper Mississippi River system, the Ozark highlands, tributaries of the Great Lakes, and the St. Lawrence River system. Sampling locations included the Upper Mississippi River drainage [Wisconsin River (WI), Mississippi River, St. Croix River (MN/WI), and Chippewa River (WI)], the Ohioan River drainage [Wabash River and White River (IN)], the Great Lakes drainage [Mississagi River (ON), Wolf River (WI) and Menominee River (WI/MI)] the St. Lawrence River drainage [St. Lawrence River (QC), St. François River (QC), Batiscan River (QC), L’Assomption River (QC), and Ottawa River (ON/QC)] (Figure 1; Tables 1 and 2). Existing mtDNA sequences from Inoue et al. (2013) were downloaded from GenBank and included in the analyses. Additionally, tissue samples from Inoue et al. (2013) from the White River in Arkansas (Mississippi Embayment) were provided by colleagues at Arkansas State University (J. Harris). For newly collected specimens, mantle tissue was nonlethally biopsied from each specimen (Berg et al., 1995), placed in 95% ethanol, and stored at −80°C.

**TABLE 1:** Collection sites, including abbreviations (code), major drainage basin, state/province, and the number of *Obovaria olivaria* samples sequenced for the mitochondrial gene COI, the number of haplotypes found at each collection location, number of unique haplotypes, the mean number of pairwise differences among haplotypes, and the mean nucleotide diversity (*π*)

Major River Drainage (Region)	Collection Site	Code	*N* sequenced @ COI	No. haplo.	No. unique haplo.	Mean No. of pairwise differences	π
St. Lawrence R.			48	4	1	0.7438	0.00144
	St. Lawrence R. (Grondines)	GRON‐STL	9	2	0	1.0000	0.00194
	St. Lawrence R. (Domaine Joly)	DOJO‐STL	9	2	0	0.4444	0.00086
	Rivière Saint Franςois	STFR‐STL	8	1	0	0.000	0.000
	Batiscan River	BAT‐STL	7	2	0	0.4762	0.00092
	Rivière L’Assomption	AS‐STL	7	4	1	1.0476	0.00203
	Ottawa R. (Lac Coulonges)	LCOU‐OTT	8	2	0	0.5000	0.00097
Great Lakes			18	4	1	1.0458	0.00203
	Mississagi R.	MISS‐ON	5	2	1	0.6000	0.00116
	Menominee R.	MEN	4	1	0	0.000	0.000
	Wolf River	WOLF	9	2	0	0.2222	0.00043
Upper Mississippi R.			31	9	4	2.3226	0.00455
	Wisconsin R.	WIPS/WIOR	11	7	2	3.2364	0.00635
	St. Croix R.	STCR	6	1	0	0.000	0.000
	Chippewa R.	CHIP	5	3	0	3.0000	0.00588
	Mississippi R. Pool 15, 24, 25	MISS‐P15/ MISS‐P24/ MISS‐P25	8	4	2	1.6071	0.00312
	Ohio River^a^	OHIOR	1	1	0	‐	‐
Mississippi Embayment			15	5	4	1.7524	0.00344
	White River^a^	WHIT‐AR	12	4	2	1.4394	0.00282
	Black River^a^	BLACK‐AR	3	2	1	3.3333	0.00646

Notes

Values presented for each drainage (shaded rows) were calculated from pools of all samples for the drainage.

^a^ From Inoue et al. (2013), sites with sample, Ohio River excluded from per‐population averages presented in Results text for small sample size.

**TABLE 2:** *Obovaria olivaria* collection sites, including abbreviations (code), major drainage basin, state/province, number genotyped, observed heterozygosity (*H*_o_), expected heterozygosity (*H*_e_), inbreeding coefficients (*F*_IS_), and private alleles for the SNP dataset

Major River Drainage (Region)	Collection Site	Code	*n*	*H*_o_	*H*_e_	Private alleles
Northern populations						
St. Lawrence R.	Rivière L’Assomption	AS‐STL	5	0.189	0.171	0
	Batiscan R.	BAT‐STL	3	0.192	0.164	0
	St. Lawrence R. (Domaine Joly)	DOJO‐STL	6	0.199	0.181	0
	St. Lawrence R. (Grondines)	GRON‐STL	10	0.195	0.193	0
	Rivière Saint Franςois	STFR‐STL	5	0.188	0.167	0
	Ottawa R. (Lac Coulonges)	LCOU‐OTT	5	0.195	0.169	0
	Ottawa R. (Lac Deschênes)	LDES‐OTT	1	0.201	0.100	0
Great Lakes populations						
Great Lakes	Wolf R.	WOLF	5	0.268	0.230	0
	Menominee R.	MEN	3	0.215	0.176	0
	Mississagi R.	MISS‐ON	4	0.216	0.192	1
Southern populations						
Upper Mississippi R.	Chippewa R.	CHIP	5	0.240	0.215	0
	St. Croix R.	STCR	5	0.235	0.209	0
	Mississippi R. Pool 15	MISS‐P15	6	0.227	0.213	0
	Mississippi R. Pool 25	MISS‐P25	1	0.243	0.122	0
	Wisconsin R. at Praire Du Sac	WPS	5	0.244	0.225	0
	Wisconsin R. at Orion	WIOR	5	0.246	0.226	0
Mississippi Embayment	White R.^a^	WHIT‐AR	4	0.216	0.178	1
Ohioan	White R.	WHIT‐IN	8	0.223	0.221	2
	Wabash R.	WABASH	7	0.219	0.211	0

Notes

Although *H*_o_ was generally greater than *H*_E_, no significant deviations from Hardy–Weinberg Equilibrium were detected (Genepop combined results across loci using Fisher's method, tests could not be performed for LDES‐OTT and MISS‐P25 for sample size). Northern, Great Lakes, and Southern population codes refer to the regions assigned for the DIYABC analyses.

^a^ Museum specimens from Inoue et al. (2013).

### 2.2 Genetic and genomic procedures

DNA was extracted using the Qiagen DNeasy Blood and Tissue Extraction Kit™ (QIAGEN) and protocol. Extracted DNA was stained with SYBR Green™ (Thermo Fisher Scientific) dye, and agarose gel electrophoresis was performed to confirm the presence of high‐quality, high molecular weight genomic DNA and concentrations were quantified using a Nanodrop spectrophotometer (Thermo Fisher model # ND‐1000).

#### 2.2.1 Sanger sequencing preparation

The cytochrome oxidase subunit 1 (COI) region of the mitochondrial genome was amplified by polymerase chain reaction (PCR) using the primers and thermocycler conditions described by Campbell et al. (2005). PCR products were verified by electrophoresis on a 1.5% agarose gel and purified using Exonuclease I (EXO, Amersham Biosciences cat. #E70073X, 10 U/ml) and Shrimp Alkaline Phosphatase (SAP, Amersham Biosciences cat. #E70092X 1 U/ml). An EXOSAP solution was created with 78 µl ddH_2_O, 2 µl EXO, and 20 µl SAP, and then, 2 µl of the mixture was added to each PCR product to remove primers, dNTPs, and other impurities, and were Sanger sequenced by Eton Bioscience (www.etonbio.com, San Diego, California) using the forward primer (Campbell et al., 2005).

#### 2.2.2 Restriction site‐associated DNA library construction and sequencing

We used the Best‐RAD protocol (Ali et al., 2016) to develop a SNP dataset. Best‐RAD employs restriction enzymes to cleave DNA into short fragments and uses high‐throughput sequencing to produce sequence data adjacent to the large number of restriction enzyme cut sites across the genome (Ali et al., 2016; Andrews et al., 2016). Genomic DNA was quantified using a PicoGreen^®^ dsDNA quantification assay (Thermo Fisher Scientific, Carlsbad, California) for precise standardization at 20 ng/µl for library preparation. Standardized DNA was digested with the restriction enzyme SbfI‐HF (New England Biolabs, Ipswich, Massachusetts), followed by ligation of synthetic oligonucleotides containing unique 8 base pair Hamming barcodes for each individual. Barcoded samples were pooled and sonicated using the Covaris S2 Adaptive Focused Acoustic Disrupter (COVARIS, Inc.) to randomly fragment barcoded DNA to an average size of 550 base pairs. RAD‐tag fragments were isolated with streptavidin beads and biotinylated groups were removed by Sbf‐1 digestion. Following digestion, a NEBNext Kit™ (New England Biolabs) was used to barcode and enrich the library for sequencing with 12 PCR cycles. Samples were sequenced on an Illumina HiSeq 4000 (Illumina Inc.) to produce 150‐bp paired‐end reads (Michigan State University Research Technology Support Facility).

### 2.3 Bioinformatics and statistical analyses

#### 2.3.1 Mitochondrial DNA barcoding dataset

**FIGURE 2:**
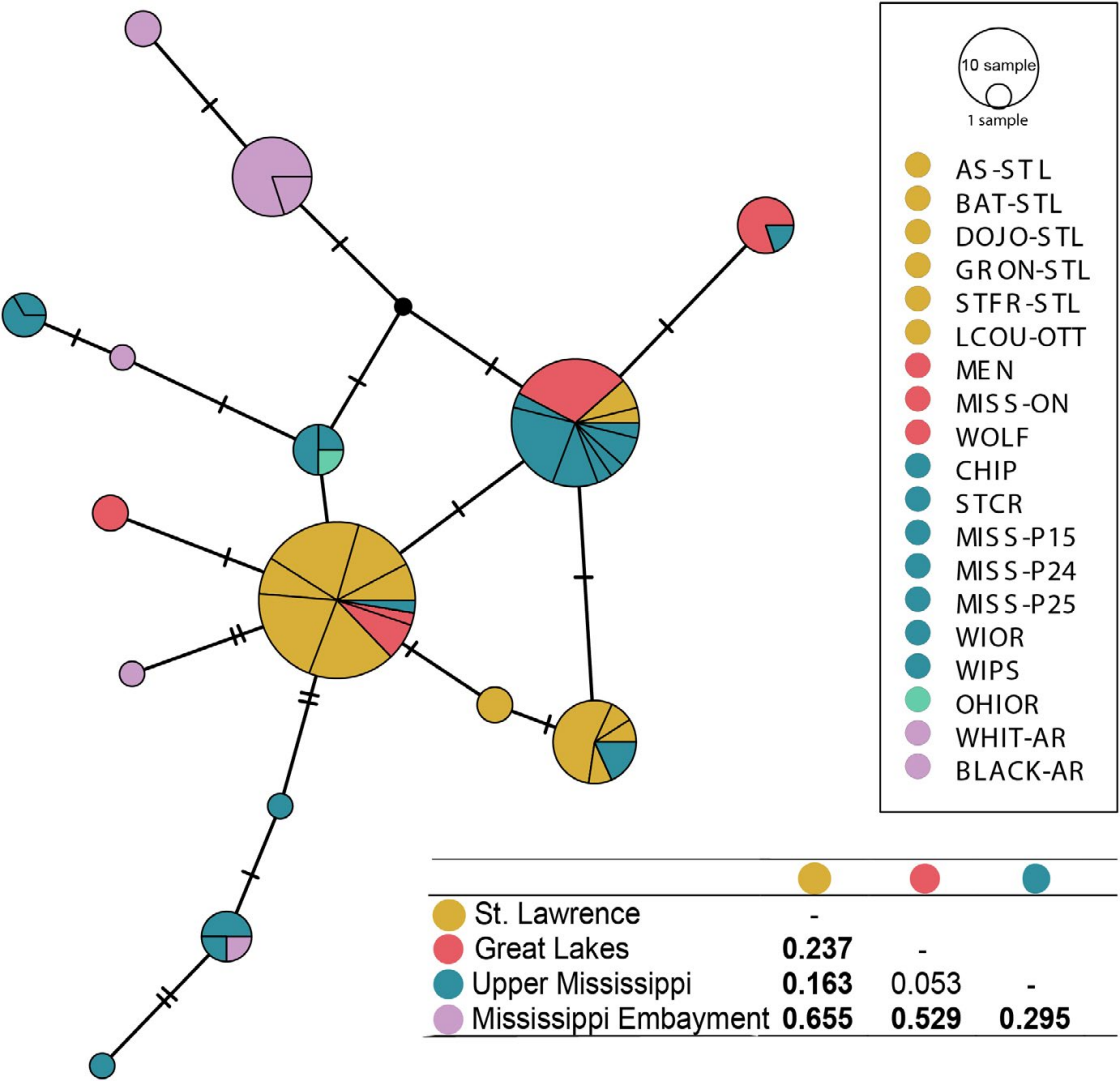
Mitochondrial COI haplotype network of 110 *Obovaria olivaria* from 19 collection locations and interdrainage Φ_ST_ values. Bold values on table indicate significant pairwise comparisons after Bonferroni correction (α = 0.0085) with 1000 permutations. Collection site codes as in Table 1

The mtDNA sequences were proofread and aligned using BIOEDIT (Hall, 1999). Additional COI sequences from the White River drainage in the southern Ozark Highlands of Arkansas and the Ohio River were included in the analyses (Inoue et al., 2013; GenBank Accession Numbers: KF035244‐KF035229). Metrics for genetic diversity (e.g., number of haplotypes, number of polymorphic sites, and nucleotide diversity – π) were calculated for each population sampled using ARLEQUIN v. 2.0 (Excoffier et al., 2005). Haplotype networks were created based on the number of nucleotide mutations between different haplotypes using POPART software and the TCS algorithm (Clement et al., 2000; Leigh & Bryant, 2015). To determine whether mussels were significantly differentiated within drainages and among sites, hierarchical analysis of molecular variance (AMOVA) was used to estimate haplotype partitioning within and among sampling locations (Excoffier et al., 1992) in Arlequin (Excoffier et al., 2005) with significance of variance components and *F*‐statistics assessed using 1,000 permutations. Pairwise Φ_ST_ values were also calculated to determine pooled sampling location differentiation at the drainage level (Figure 2).

#### 2.3.2 RAD‐seq generated SNP dataset

The quality of Illumina reads was assessed using FASTQC (Andrews, 2010), and the data were cleaned, processed, and called using Stacks v2.53 (Catchen et al., 2013). To demultiplex and clean reads, we used process_radtags (parameters ‐c, ‐q, ‐r, ‐‐best‐rad, others default). Because there is no reference genome for *O*. *olivaria*, *de novo* locus assembly and SNP calling were performed using denovo_map.pl, with removal of PCR duplicates (parameters: ‐‐rm‐duplicates, ‐‐paired, ‐‐time‐components, others default). We used vcftools (Danecek et al., 2011) to create a final dataset with SNPs present in ≥75% of individuals and present in all sampling locations. We required a minimum minor allele frequency of 0.02 to reduce the impact of low‐frequency alleles and possible genotyping error (Rochette et al., 2019, but see Supplementary materials Appendix S1 and S2 for results with all SNPs) and thinned the dataset to retain a single SNP per RAD‐tag locus to remove tightly linked sites. Individuals with >50% missing data were removed. Unless otherwise stated, all remaining analyses were conducted in R version 4.0.3 (R Core Team, 2018). The R package *radiator v1*.*1*.*9* was used for import and data format conversion (Gosselin, 2017). To confirm that the removal of rare alleles did not impact the genetic structure observed in the dataset, a principal component analysis (PCA) was performed. First, the vcf file was converted to a *genind* object in *adegenet v2*.*1*.*3*, and then, the function *tab* was used to replace missing data with mean allele frequency (Jombart & Ahmed, 2011). The PCA was conducted in R package using *ade4 v1*.*7*.*16* (Dray & Dufour, 2007) with the function *dudi*.*pca* (see Appendix S3).

To assess genetic diversity, we calculated observed and expected heterozygosity and the inbreeding index (*H_o_*_,_ *H_e_*, *F*_IS,_ and private alleles) of SNPs for each sampling location using the *radiator* package's *summary_rad* and *private_alleles* functions (Gosselin, 2017). The *radiator* package's *write_genepop* function (Gosselin, 2017) was used to convert the vcf to genepop format (Raymond & Rousset, 1995; Rousset et al., 2020), and Hardy–Weinberg calculations were conducted using the R package *genepop* v1.1.7 with the function *test_HW* (Rousset et al., 2020). We tested for significant Hardy–Weinberg deviations for each locus within each population and across populations (combining results across populations using Fisher's Method) and overall for each population (combining results across SNPs using Fisher's Method). Genetic structure was assessed using several complementary methods. Individual‐level genotype clustering was first determined using the *snmf* function in the R package *LEA v3*.*2*.*0* (Frichot & François, 2015) with *K* (number of genetic populations) determined by the value producing the lowest cross‐entropy (Frichot & François, 2015). Plots of *snmf* qmatrix results were made with the R package *ggplot2 v3*.*3*.*0* (Frichot & François, 2015; Villanueva & Chen, 2019). We also performed a discriminant analysis of principal components (DAPC) using *adegenet v2*.*1*.*3* (Jombart & Ahmed, 2011), with the optimal number of clusters (*K*) determined by the value producing the lowest Bayesian information criterion from *k*‐means clustering (*find*.*clusters* function). The DAPC scatterplot was created with *ggplot2* and *ggforce v0*.*3*.*1* (Pederson, 2019; Villanueva & Chen, 2019). Pairwise measures of genetic differentiation (*F*_ST_) were computed in the R package *StAMPP v1*.*6*.*1* (Pembleton et al., 2013), with significance determined using 1000 bootstrap replicates. We used AMOVA in Arlequin v3.5 as above (Excoffier et al., 2005) to estimate genotype partitioning between and among sampling locations and genetic clusters determined by the DAPC in *adegenet* (Jombart & Ahmed, 2011).

To assess the location of potential geographic barriers to gene flow exist with the SNP dataset, Monmonier's algorithm was used to find the boundaries of maximum differences between contiguous polygons in a tessellation and to detect geographic locations of barriers to gene flow among genotypes (Monmonier, 1973). The Monmonier's algorithm was executed in R package *adegenet* using the *monmonier* function with pairwise *F*_ST_ and site coordinates (Jombart & Ahmed, 2011; Manni et al., 2004; Monmonier, 1973).

To test for isolation‐by‐distance, we determined geographic river distances between sites in R package *riverdist v0*.*15*.*3*. Prior to *riverdist* calculations, shapefiles from DivaGIS v7.5 (Villordon, 2019) for North American and Canadian rivers were cropped and merged in ArcMap v10.8 to only include relevant segments in the shapefile. The outline of the Great Lakes was used to create a line segment around the Great Lakes for distance calculations (ESRI, 2018; Villordon, 2019). The function *cleanup* in *riverdist* (Tyers, 2017) was used to measure straight‐line distances (snapping to closest point in *riverdist*) to account for lakes and short overland distances between drainages (i.e., Wisconsin River and Fox/Wolf River into the Great Lakes and the Lake Nipissing drainage in the Great Lakes and Ottawa River/St. Lawrence River drainages). After assigning the Mississippi (above STCR site) as the river mouth in *riverdist*, the *detectroute* function was used to measure the distances between points on the river network segment (Tyers, 2017). Mantel tests for isolation‐by‐distance were performed in R package *adegenet* with the function *mantel*.*randtest* with 999 permutations using pairwise *F*_ST_ and pairwise river distances (Jombart & Ahmed, 2011). We excluded sites with only one sampled individual (MISS‐P25, LDES‐OTT) and one site (DOJO‐STL) because of channel braiding, which can produce incorrect distance calculations (Tyers, 2017).

Finally, we were interested in using the SNP data to test various demographic scenarios that might explain genetic diversity and structure among the major regional populations. We were interested in varying patterns of divergence that reflect postglacial expansion from likely glacial refugia from southern into northern populations. Frequently, freshwater mussels exhibit a stepping‐stone model of postglacial colonization, entering the Great Lakes from the Mississippi or Wabash refugia and then expanding northward (Beaver et al., 2019; Elderkin et al., 2007, 2008; Mathias et al., 2018). For model testing, we conducted approximate Bayesian computation with random forests using DIYABC RF v1.0 and the R package *diyabcGUI* v 1.0.14 (Collin et al., 2021). We grouped mussels into three regional population clusters identified by DAPC: (1) *Southern* populations including the Upper Mississippi River basin and Mississippi Embayment, (2) *Great Lakes* basin populations, (3) *Northern* populations in St. Lawrence River basin (see Table 2 for population assignments to regions). We first used DIYABC with the mitochondrial dataset to determine reasonable upper bounds on prior distributions for modeling with the SNP dataset. Default mutation model parameters were used to model COI sequence data, and maximum effective population sizes and generation times were varied to determine the best fit for the SNP dataset. We ran the COI testing models with 140,000 iterations and validated models with 500 random forests. Based on the effective population size estimates from mtDNA sequence data, in the SNP data, we set uniform prior distributions for effective population size (henceforth, N_e_) and time to be a maximum of 2.5 million individuals for all regional populations and set a maximum divergence time of 50,000 generations. We also examined models constraining N_e_ to 1/10^th^ this value (250k) to examine sensitivity to this prior. We tested seven potential scenarios of postglacial colonization (Appendix S4), including a null model and variations of a bottleneck pre‐ and postdivergence for our populations. (1) First, we chose a null model with a single divergence event for the Southern, Great Lakes, and Northern populations from a single common ancestor at the same time point. (2) Next, we simulated a model with divergence between Southern and Northern populations with a single admixture event forming the Great Lakes (prior on admixture proportion: admixture rate of 0.05–0.95 with a uniform distribution, default setting). Three related models were intended to model stepping‐stone colonization of postglacial colonization using multiple divergence times: (3) Southern populations splitting to form the Great Lakes and then a subsequent Great Lakes split leading to the formation of the Northern populations, (3a) one of our stepping‐stone models had with constant population sizes throughout, (3b) one with a variation in population sizes at the Great Lakes split only to simulate a potential bottleneck, (3c) one with variation in population sizes at both the Great Lakes split and Northern split to simulate two potential bottlenecks. (4) A fourth model simulated a divergence between the Southern populations and the Great Lakes with admixture (prior of 0.05–0.95 with a uniform distribution) following with the formation of the Northern populations from the Great Lakes ancestor. (5) Lastly, we simulated a model where the Northern populations and Southern populations split from a common ancestor and then Northern populations split to form the Great Lakes. ABC modeling was conducted from command line on an HPC cluster with 140,000 (20,000 simulations per model for model choice) simulations. A random forest estimation was then used to determine the best model fit, and parameter estimates from the best fit model were determined from 120,000 simulations using random forest with 500 trees for each parameter, prediction of models and parameter estimates was with 500 trees for each parameter estimated.

## 3 RESULTS

### 3.1 Mitochondrial DNA dataset

A 515‐bp fragment of the COI mtDNA gene was sequenced from 112 individuals collected from 16 locations across 4 major drainages: the St. Lawrence River drainage (6 sites), the Laurentian Great Lakes watershed (3 sites), Upper Mississippi River drainage (5 sites), and Mississippi Embayment (White and Black Rivers, 2 sites; Table 1). The sequencing identified 11 new COI haplotypes (GenBank Accession Numbers: MN413582‐MN413592), for a total of 15 haplotypes comprising new and previously published sequences (Figure 2). The most frequent haplotypes were shared among collection sites and drainages (Figure 2). Most of the 10 haplotypes unique to any drainage were found south of the Great Lakes (four each in the Upper Mississippi River and Mississippi Embayment regions). Nucleotide diversity (π) per population was highest among the Upper Mississippi River sites (0.0038 ± 0.0015 SE) and Mississippi Embayment sites (0.0046 ± 0.0018 SE), and lower in Great Lakes sites (0.0005 ± 0.0003 SE) and St. Lawrence River sites (0.0011 ± 0.0003 SE); similar patterns were apparent for the average pairwise differences (Figure 2).

Genetic differentiation was evident among both drainages (AMOVA *F*_CT_ = 0.27, *p* < .001) and individual collection sites (AMOVA *F*_ST_ = 0.45; *p* < .001) (Table 3). Pairwise genetic differentiation among collection sites (Φ_ST_) was generally low within each drainage and higher between drainages although Bonferroni‐corrected pairwise differentiation was only consistently observed with the White River site (Figure 2), likely due to small sample sizes per site. Pairwise Φ_ST_ among drainages were significant except for the Great Lakes and Upper Mississippi River drainages comparison (Figure 2).

**TABLE 3:** Analysis of molecular variance (AMOVA) for *Obovaria olivaria* using COI mtDNA sequence data

Source of variation	*df*	Sum of squares	Percentage of variation	*F* Statistics	*p*
Among regions	3	27.375	27.45%	*F*_CT_	.275***
Among sampling locations within regions	12	20.229	17.16%	*F*_SC_	.237***
Within sampling locations	96	53.245	55.38%	*F*_ST_	.446***
Total	111	100.848			

Notes

Four regions are: St. Lawrence R., Great Lakes, Mississippi R., White R. (Mississippi Embayment).

*** *p *< .001.

### 3.2 SNP dataset

Illumina sequencing of the *Obovaria olivaria* BestRAD libraries yielded an average of 2,041,264 (1,596,292 SD) paired‐end reads per sample. The final filtered dataset included 1,237 polymorphic SNPs present in the 93 individuals from 19 collection locations (Table 2), with an average SNP depth of 19.15 (Appendix S5), <30% missing data and a minor allele frequency >0.02.

There were clear spatial patterns in diversity in the population genomics dataset that were consistent with COI sequences, with northern and eastern populations having reduced heterozygosity compared to populations from south of the Great Lakes, with diversity especially low in the St. Lawrence River and highest in the Wolf and Mississippi River sites (Table 2; Appendix S6). Observed heterozygosity was higher than expected heterozygosity across all collection locations (Table 2), but no significant deviations from Hardy–Weinberg equilibrium were detected in any population (Table 2). Four private alleles were found in the SNP dataset, and all were found south of the St. Lawrence (Table 2).

**FIGURE 3:**
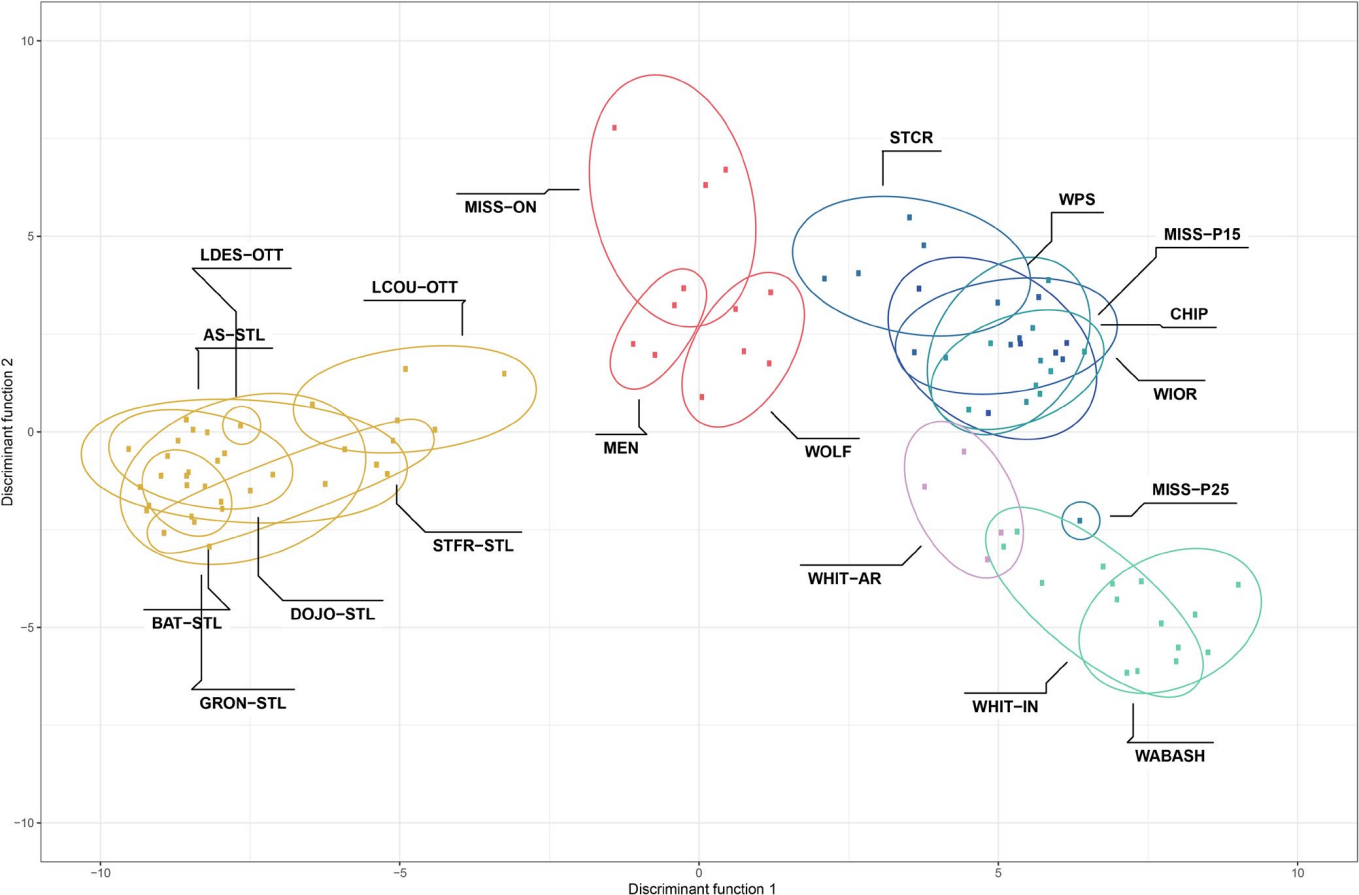
Discriminant analysis of principal components of *Obovaria olivaria* populations. Ellipses represent 95% confidence intervals for each group

**FIGURE 4:**
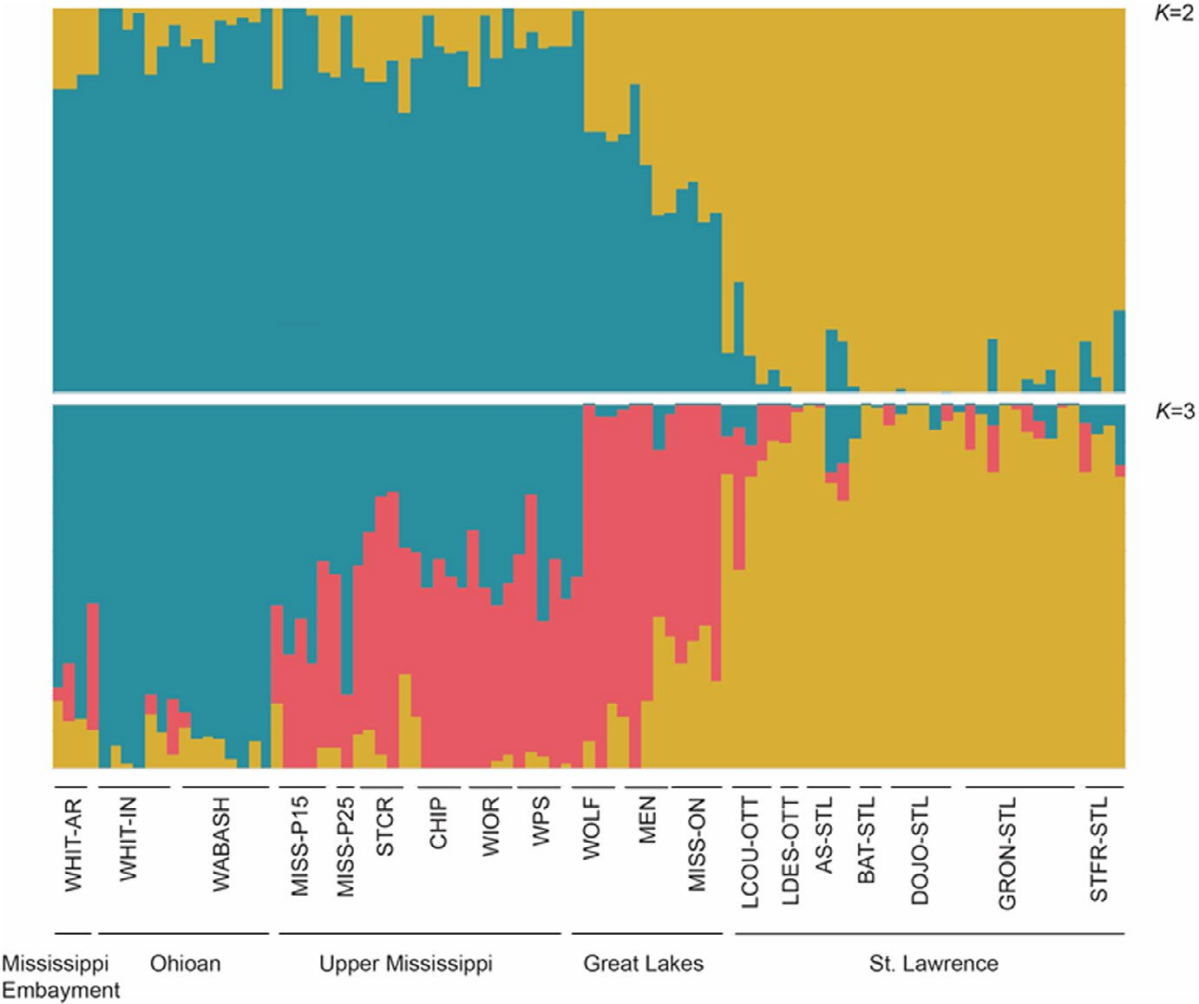
Results from admixture analyses in R package LEA for both *K *= 2 and *K *= 3

The DAPC and snmf analyses of the SNP dataset both revealed two major clusters, but there was clear admixture at spatially intermediate sites (Figure 3; Appendix S7). For the DAPC, *K* = 2 was identified as the optimal number of ancestral populations, with St. Lawrence and Ontario River populations loading negatively on axis 1 and the Upper Mississippi, Mississippi Embayment, and Ohioan River populations loading positively, and with the Great Lakes populations intermediate between the two groups; axis 2 discriminated among sites within the Upper Mississippi, White, and Ohioan Rivers. For snmf, *K *= 2 had the lowest cross‐entropy, indicating two ancestral population groupings: (1) all the individuals from the St. Lawrence River drainage, (2) all individuals from the Mississippi watershed, Great Lakes watershed, the White River, Wabash, and White River in Illinois, but as above the Great Lakes mussels were intermediate (Figure 4). We repeated the snmf analysis for *K *= 3, which partly separated sites from the Great Lakes region; however, admixture among ancestral populations was still apparent. AMOVA showed significant genetic differentiation among the two regional clusters (*F*_CT_ = 0.11, *p* < .001) (Table 4), as well as among sites within clusters (*F*_SC_ = 0.05, *p* < .001) and among sites overall (*F*_ST_ = 0.16, *p* < .001).

**TABLE 4:** Analysis of molecular variance (AMOVA) for *Obovaria olivaria* using SNP data completed in Arlequin with 1000 permutations

Source of variation	Sum of squares deviation	Percentage of variation	*F*‐statistics	*p*
Among clusters	1589.99	10.9%	*F*_CT_	.11***
Among populations within clusters	3459.01	4.8%	*F*_SC_	.05***
Within populations	19703.10	84.3%	*F*_ST_	.16***
Total	24752.09			

Notes

The two clusters are (1) St. Lawrence and Ottawa River sites; (2) Great Lakes, Upper Mississippi R., White R. (Mississippi Embayment), and Ohioan drainage collection locations.

*** *p* < .001.

**FIGURE 5:**
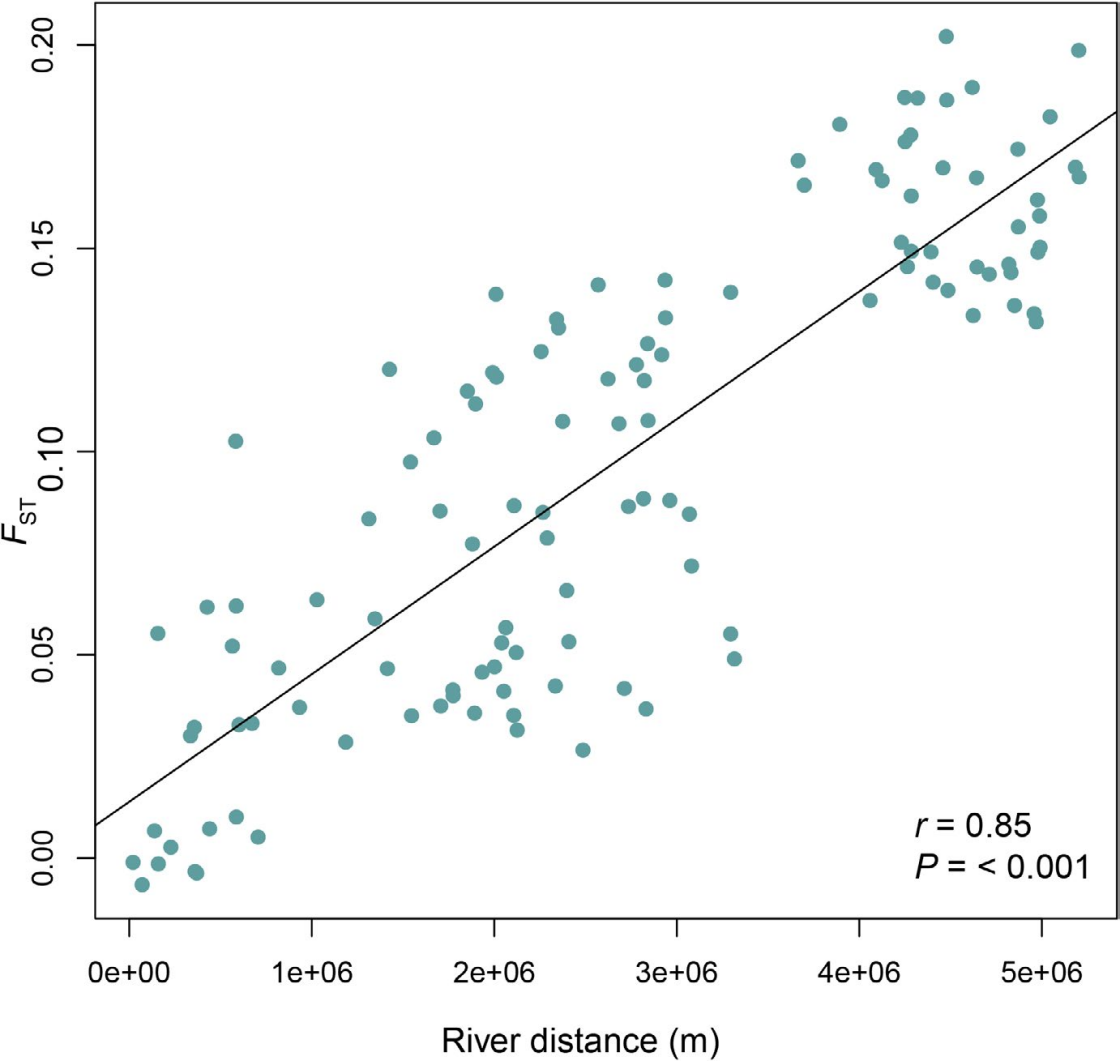
Mantel test for pairwise river distances between sampling sites and pairwise estimates of *F*_ST_ from SNP data

When differentiation among pairs of populations was examined, there was significant low to moderate genetic differentiation (*F*_ST_) among most collection sites (Appendix S8), with values ranging from *F*_ST_ = 0.00–0.20. Monmonier's algorithm detected a barrier to gene flow between the St. Lawrence/Ottawa collection locations and the remaining collection locations at Lake Erie (42.0894, −81.70649), consistent with the clusters identified by DAPC. However, also consistent with the DAPC and snmf clustering that suggested admixture among ancestral populations via intermediate sites, Mantel tests showed a strong isolation‐by‐distance relationship between pairwise *F*_ST_ and river distances at the range‐wide scale (Mantel *r* = 0.85; *p* < 0.001; Figure 5).

The DIYABC scenario choice determined that the null model (simultaneous divergence of three regional populations) was the best fit model for the SNP and mitochondrial datasets, suggesting limited power in this system to resolve any directional colonization history. However, DIYABC models did consistently show that relative N_e_ was sharply reduced in the Northern populations of *Obovaria olivaria* (Table 5; Appendix S9). DIYABC models were sensitive to prior parameter settings and although Northern N_e_ was always well‐estimated, *N*_e_ from Southern and Great Lakes populations had broad posterior distributions (Appendix S9). We thus urge caution when interpreting results as real‐world values but suggest the variation in N_e_ from population to population is more indicative of general trends in relative effective size (Table 5; Appendix S9). The median generation time since population split was 26,498 generations in the upper bound model (although as for N_e_ the posterior was broad), but similar to geologic estimates of the Great Lakes formation of ~14,000 YBP (Larsen, 1985) in the lower bound model (13,166 generations). Overall, we were unable to precisely estimate parameters in this dataset with DIYABC, but results are consistent with summary statistics presented above that suggest substantially reduced genetic diversity in *O*. *olivaria* from the northern St. Lawrence River populations.

**TABLE 5:** Prior and posterior distributions for effective population sizes (Ne) and generation time (t) for *Obovaria olivaria* genetic clusters inferred from the DAPC analysis

Parameter	Population	Prior distributions				Posterior parameter estimates	
		Minimum	Maximum	Distribution	Median	95% credible interval	
*N_e_*	Southern	10	250,000	Uniform	175,101	65,200	247,255
*N_e_*	Great Lakes	10	250,000	Uniform	112,264	21,129	236,280
*N_e_*	Northern	10	250,000	Uniform	9,863	1,507	45,150
*t_1_*		10	50,000	Uniform	13,166	3,163	36,452
*N_e_*	Southern	10	2,500,000	Uniform	1.80 × 10^6^	643,676	2.45 × 10^6^
*N_e_*	Great Lakes	10	2,500,000	Uniform	1.10 × 10^6^	228,206	2.32 × 10^6^
*N_e_*	Northern	10	2,500,000	Uniform	63,420	19,221	220,130
*t_1_*		10	50,000	Uniform	26,498	7,717	46,939

Notes

Two sets of upper prior bounds on *N*_e_ are presented, indicating the sensitivity of absolute posterior distributions to selection of priors, while relative patterns are similar. The Southern populations comprise the Upper Mississippi River, the Ohioan and the Mississippi Embayment sites, the Great Lakes populations comprise the Great Lakes drainage sites, and the Northern populations comprise the St. Lawrence River drainage sites.

## 4 DISCUSSION

Our combined analysis of mitochondrial and RAD‐tag sequences in *O*. *olivaria* provides evidence for ongoing or recent connectivity driven by spatial separation within and between regions, which also exhibit differences in genetic diversity. The patterns of range‐wide isolation‐by‐distance presented here imply that *O*. *olivaria* dispersal is distance‐limited, but such limitations occur at large scales and suggest the potential for long‐distance movement on host fish. We also find that *O*. *olivaria* exhibits notably reduced genetic diversity in northern sites (the St. Lawrence and Ottawa Rivers), which is especially concerning given the endangered status of these populations.

In the mitochondrial DNA dataset, diversity declined from south to north, with reduced haplotype and nucleotide diversity in the Great Lakes and St. Lawrence drainages and the greatest number of haplotypes and unique haplotypes is found in the Mississippi River drainage. The White (AR) and Black River individuals had 4 unique haplotypes out of 5 haplotypes, which is consistent with other phylogeographic studies on freshwater taxa from this region that display similar endemism (Crandall, 1998; Mayden, 1985; Vaughn et al., 1996; Zanatta & Murphy, 2008). Despite the presence of some unique haplotypes in the southern populations and significant regional Φ_ST_ values, the COI data indicate no extensive phylogeographic barriers across the *O*. *olivaria* range.

Results from the SNP data largely parallel those from the mitochondrial sequences. Heterozygosity was greatest in the Mississippi and Great Lakes drainage sites and declined toward the St. Lawrence collecting sites (Table 2). As anticipated, the *O*. *olivaria* SNP dataset revealed more fine‐scale genetic structure than analyses of the mtDNA dataset, indicating the presence of two major population clusters, one predominantly in the St. Lawrence River drainage and one in the Upper Mississippi, Mississippi Embayment, and Ohioan, and some weaker separation of mussels in the Great Lakes drainages. Weak structure was seen between the Mississippi and Great Lakes populations, which historically were not connected for decades due to changes in the configuration of the Great Lakes after the Pleistocene and were then subsequently artificially connected by canals. Although the Great Lakes sampling locations appear to have some unique genetic variation, with one private allele found in the Mississagi River location and high genetic diversity at the Wolf River site (Table 2; Figure 4), these populations are for the most part genetically intermediate between the more southern and northern localities and provide evidence of historical connectivity across the species range.

Patterns of genetic structure in freshwater mussel species often reflect changes in the patterns of hydrologic connections in the Great Lakes at the end of the last glacial period (Elderkin et al., 2007, 2008; Mathias et al., 2018). For example, at the end of the Pleistocene glaciation (~6000 to ~4500 YBP), the Great Lakes and the St. Lawrence River drainages remained connected near North Bay, Ontario (Larsen, 1985; Teller, 1985), but these connections were later severed (~4500 YBP) (Larsen, 1985). This change in the configuration of the Great Lakes drainage left only one remaining outlet to the St. Lawrence River drainage, via Lake Erie and Lake Ontario. A disruption of gene flow in the Great Lakes would be consistent with the location of the genetic barrier at Lake Erie inferred by the Monmonier's algorithm in *O*. *olivaria*. Further, the declining genetic diversity from south to north supports the hypothesis that the Mississippi River drainage may have served as a glacial refuge for *O*. *olivaria* populations. We were thus interested in testing whether patterns of structure and declining south‐to‐north diversity are related to a stepping‐stone model of postglacial colonization (Hewitt et al., 2018; Kimura & Weiss, 1964; Mathias et al., 2018; Zanatta & Harris, 2013). While a more complex demographic history for *O*. *olivaria* than one of ongoing distance limited dispersal among contemporary populations is likely, DIYABC was unable to distinguish scenarios of postglacial colonization. This is perhaps due to limitations in software's ability to simulate ongoing migration or complex population structure within our broad regional populations, especially in the south (Collin et al., 2021). Our data are thus most consistent with a model of population structure involving contemporary regional populations undergoing regular distance‐limited genetic exchange, which has likely complicated historical phylogeographic signatures. Given this inability to clearly distinguish among models and the observed sensitivity of parameters to N_e_ priors, we urge caution in interpreting DIYABC results as “real‐world” estimates. However, models consistently support the conclusion that the St. Lawrence and Ottawa Rivers harbor substantially smaller population sizes than other remaining regions sampled for this study. These Canadian *O*. *olivaria* populations are endangered (COSEWIC, 2011), and density in the Ottawa River is relatively low compared to other unionid species in the area (Martel et al., 2011). In contrast, N_e_ estimates for the Southern (Upper Mississippi, Mississippi Embayment, and Ohio Rivers) and Great Lakes regions were high (>1 million individuals in our upper bound model), suggesting that these populations have retained high levels of ancestral genetic diversity and may not have suffered the genetic impacts of any population declines.

Genetic differentiation and diversity of *O*. *olivaria* largely mirrors that seen in confirmed and potential host fish with analogous distributions. Mitochondrial haplotypes of *O*. *olivaria* host fish *A*. *fulvescens* reveal patterns of limited genetic structure (DeHaan et al., 2006; Ferguson & Duckworth, 1997). Mitochondrial (DeHaan et al., 2006; Ferguson & Duckworth, 1997) and microsatellite data from *A*. *fulvescens* (Welsh et al., 2008) show a pattern of genetic structure consistent with our *O*. *olivaria* SNP dataset. Welsh et al. (2008) also found *F*_ST_ for *A*. *fulvescens* among the Great Lakes drainage sampling locations (including samples from the Wolf, Menominee, and Mississagi rivers) was low, but significant (0.02–0.05, *p* < .002), and *F*_ST_ among St. Lawrence River sampling locations was also low, but significant (0.03–0.04, *p* < .002). The lack of discrete genetic structure in *O*. *olivaria* is likely linked to *A*. *fulvescens* and other host fish and therefore prioritizing management of habitats where both species coexist will likely ensure greater conservation success.

### 4.1 Conservation Recommendations

The results of this study emphasize the importance of riverscape genetics in identifying historically connected populations (Davis et al., 2018). These data indicate that gene flow has likely been maintained across the *O*. *olivaria* range by utilizing the movement of their host fish and via movement through stream capture or isostatic rebound events during the changes in the configuration of the Great Lakes following deglaciation. However, *O*. *olivaria* are relatively long‐lived (up to ~50 years, D. Zanatta pers. obs.), have relatively long generation times (~7–14 years, COSEWIC, 2011), and have large population sizes in considerable portions of their range (Table 5), so it may take decades to centuries for any contemporary barriers to gene flow to result in detectable divergence of recently isolated populations (Hoffman et al., 2017). Therefore, routine genetic monitoring of at‐risk populations may be advisable. Further, in both the mitochondrial and SNP data, the St. Lawrence and Ottawa river populations, which are considered endangered, exhibit the lowest genetic diversity and N_e_, suggesting that these populations should be given priority for conservation and restoration efforts. Although no discrete lineages are present in *O*. *olivaria* across its range, the clinal population structure and diversity leads us to recommend that if supplementation or propagation is required to enhance diversity of threatened or endangered populations, the use of genotypes from adjacent populations would be the optimal strategy. It is important to note that while this study focuses on neutral genetic variation that shows evidence of considerable admixture in many parts of the distribution of *O*. *olivaria*, adaptive differences may exist between populations. The numbers of SNPs here are insufficient to detect such adaptation, but as whole‐genome sequencing becomes more readily accessible examining genetic markers that may be under selection could elucidate evolutionary differences among populations beyond those seen here (Funk et al., 2012). Finally, management plans should also take into consideration the genetic structure and diversity of not only *O*. *olivaria* but also that of *A*. *fulvescens* and *S*.* platorynchus*, as the continued persistence of *O*. *olivaria* is intrinsically dependent on these sturgeon host fishes.

## CONFLICT OF INTEREST

None declared.

## AUTHOR CONTRIBUTION

**Jamie R. Bucholz:** Conceptualization (supporting); Data curation (lead); Formal analysis (lead); Investigation (lead); Methodology (lead); Validation (lead); Visualization (lead); Writing – original draft (lead); Writing – review & editing (lead). **Nicholas M. Sard:** Data curation (supporting); Methodology (equal); Resources (equal); Supervision (supporting); Writing – original draft (supporting); Writing – review & editing (supporting). **Nichelle M. VanTassel:** Data curation (supporting); Writing – review & editing (supporting). **Jeffrey D. Lozier:** Resources (equal); Software (equal); Supervision (supporting); Validation (supporting); Visualization (supporting); Writing – original draft (supporting); Writing – review & editing (supporting). **Todd J. Morris:** Conceptualization (supporting); Data curation (supporting); Funding acquisition (supporting); Project administration (supporting); Writing – review & editing (supporting). **Annie Paquet:** Conceptualization (supporting); Data curation (supporting); Funding acquisition (supporting); Investigation (supporting); Project administration (supporting). **David T. Zanatta:** Conceptualization (lead); Data curation (equal); Formal analysis (equal); Funding acquisition (lead); Investigation (equal); Methodology (equal); Resources (lead); Software (equal); Supervision (lead); Validation (lead); Visualization (lead); Writing – original draft (equal); Writing – review & editing (equal).

## Supporting information

Supplementary Material

## Data Availability

The vcf file used for analyses is available at: https://doi.org/10.5061/dryad.sqv9s4n5g. Raw sequence reads are available on the Sequence Read Archive (SAMN20978488‐ SAMN20978580; BioProject: PRJNA757768).
