# Supplementary material for: RAD‐tag and mitochondrial DNA sequencing reveal the genetic structure of a widespread and regionally imperiled freshwater mussel, Obovaria olivaria (Bivalvia: Unionidae)
Source: Ecol Evol. 2022 Jan 26;12(1):e8560. doi: 10.1002/ece3.8560 (PMC8794720; doi:10.1002/ece3.8560)
Supplement: Supplementary file 1 — Supplementary Material [file ECE3-12-e8560-s001.docx]

**SUPPLEMENTAL INFORMATION FOR**

**RAD-tag and mitochondrial DNA sequencing reveal the genetic structure of a widespread and regionally imperiled freshwater mussel, *Obovaria olivaria* (Bivalvia: Unionidae)**

Jamie R. Bucholz, Nicholas M. Sard, Nichelle M. VanTassel, Jeffrey D. Lozier, Todd J. Morris, Annie Paquet, David T. Zanatta

**Appendix S1)** *Obovaria olivaria* collection sites, including abbreviations (code), major drainage basin, state/province, number genotyped, observed heterozygosity (*H*_o_), expected heterozygosity (*H*_e_), and private alleles for the SNP dataset without MAF filtering (1,415 SNPs).

| **Major River Drainage (Region)** | **Collection Site** | **Code** | ***n*** | | ***H*_o_** | ***H*_e_** | **Private alleles** |
| --- | --- | --- | --- | --- | --- | --- | --- |
| **St. Lawrence R.** |  |  |  | |  |  |  |
|  | Rivière L’Assomption | AS-STL | 5 | | 0.133 | 0.122 | 2 |
|  | Batiscan R. | BAT-STL | 3 | | 0.137 | 0.117 | 0 |
|  | St. Lawrence R. (Domaine Joly) | DOJO-STL | 6 | | 0.143 | 0.129 | 1 |
|  | St. Lawrence R. (Grondines) | GRON-STL | 10 | | 0.138 | 0.138 | 7 |
|  | Rivière Saint Franςois | STFR-STL | 5 | | 0.136 | 0.120 | 2 |
|  | Ottawa R. (Lac Coulonges) | LCOU-OTT | 5 | | 0.138 | 0.118 | 3 |
|  | Ottawa R. (Lac Deschênes) | LDES-OTT | 1 | | 0.143 | 0.072 | 0 |
| **Great Lakes** |  |  |  | |  |  |  |
|  | Wolf R. | WOLF | 5 | | 0.199 | 0.171 | 27 |
|  | Menominee R. | MEN | 3 | | 0.157 | 0.130 | 1 |
|  | Mississagi R. | MISS-ON | 4 | | 0.157 | 0.139 | 5 |
| **Upper Mississippi R.** |  |  |  | |  |  |  |
|  | Chippewa R. | CHIP | 5 | 0.180 | | 0.162 | 18 |
|  | St. Croix R. | STCR | 5 | 0.174 | | 0.155 | 16 |
|  | Mississippi R. Pool 15 | MISS-P15 | 6 | 0.169 | | 0.159 | 17 |
|  | Mississippi R. Pool 25 | MISS-P25 | 1 | 0.180 | | 0.090 | 4 |
|  | Wisconsin R. at Praire Du Sac | WPS | 5 | 0.190 | | 0.174 | 26 |
|  | Wisconsin R. at Orion | WIOR | 5 | 0.187 | | 0.172 | 32 |
| **White R. –** **Mississippi Embayment** |  |  |  | |  |  |  |
|  | White R.* | WHIT-AR | 4 | | 0.167 | 0.138 | 19 |
| **Ohioan** |  |  |  | |  |  |  |
|  | White R. | WHIT-IN | 8 | | 0.172 | 0.171 | 56 |
|  | Wabash R. | WABASH | 7 | | 0.170 | 0.165 | 65 |

**Appendix S2)** Histogram of minor allele frequencies for dataset without (A) and with (B) a MAF filter applied.

**
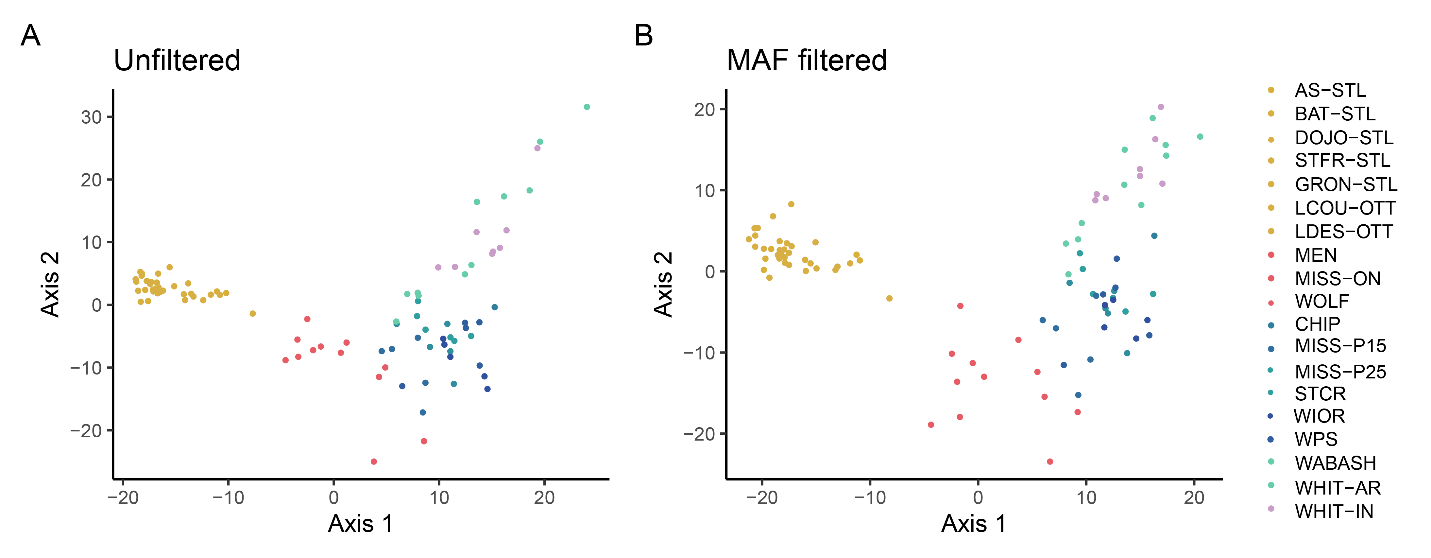
**

**Appendix S3)** PCA of *O. olivaria* sampling locations without (1,415 SNPs, A) and with (1,237 SNPs, B) a MAF filter applied, with null values replaced as the mean allele frequency.

.


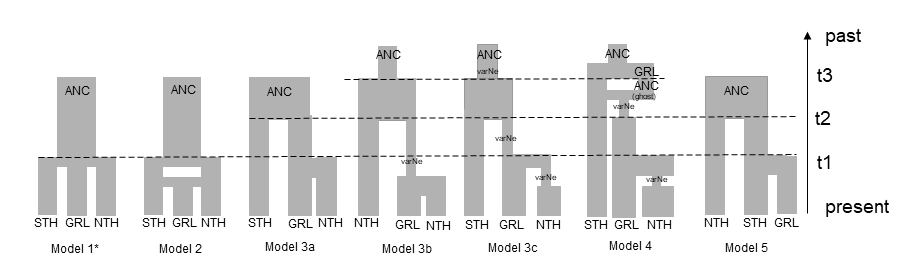


**Appendix S4)** Conceptual diagram of DIYABC scenarios, * indicates the best fit model.

**Appendix S5)** Histogram of the mean depth per site per individual for the SNP dataset.

**
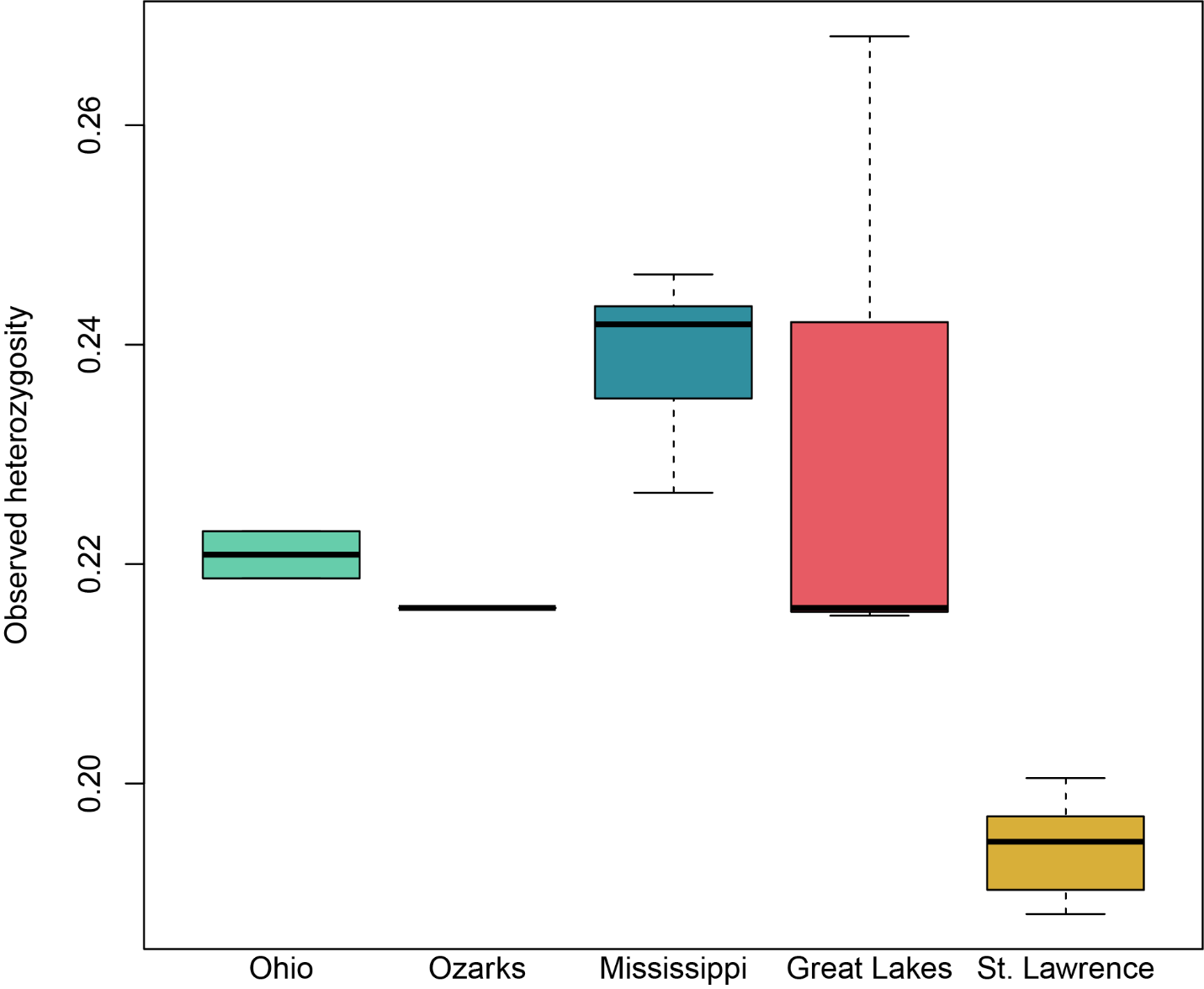
**

**Appendix S6)** Boxplot of *O. olivaria* observed heterozygosity by drainage

**Appendix S7)** Estimating number of ancestral/genetic populations (K) using cross-entropy

**Appendix S8)** Pairwise *F*_ST_ values among collection locations calculated using SNP data from *O. olivaria*. Values with grey shading are not statistically different from zero (i.e., no differentiation) in R package STAMPP. Collection site codes as in Table 1.

|  | AS-STL | BAT-STL | DOJO-STL | GRON-STL | STFR-STL | LCOU-OTT | WOLF | MEN | MISS-ON | CHIP | STCR | MISS-P15 | WPS | WIOR | WHIT-AR | WHIT-IN |
| --- | --- | --- | --- | --- | --- | --- | --- | --- | --- | --- | --- | --- | --- | --- | --- | --- |
| AS-STL | - | - | - | - | - | - | - | - | - | - | - | - | - | - | - | - |
| BAT-STL | 0.007 | - | - | - | - | - | - | - | - | - | - | - | - | - | - | - |
| DOJO-STL | 0.019 | 0.000 | - | - | - | - | - | - | - | - | - | - | - | - | - | - |
| GRON-STL | 0.000 | 0.000 | 0.000 | - | - | - | - | - | - | - | - | - | - | - | - | - |
| STFR-STL | 0.055 | 0.049 | 0.071 | 0.055 | - | - | - | - | - | - | - | - | - | - | - | - |
| LCOU-OTT | 0.062 | 0.052 | 0.068 | 0.062 | 0.103 | - | - | - | - | - | - | - | - | - | - | - |
| WOLF | 0.121 | 0.124 | 0.131 | 0.133 | 0.142 | 0.130 | - | - | - | - | - | - | - | - | - | - |
| MEN | 0.107 | 0.117 | 0.121 | 0.108 | 0.127 | 0.125 | 0.032 | - | - | - | - | - | - | - | - | - |
| MISS-ON | 0.115 | 0.119 | 0.132 | 0.118 | 0.139 | 0.120 | 0.064 | 0.037 | - | - | - | - | - | - | - | - |
| CHIP | 0.144 | 0.136 | 0.149 | 0.155 | 0.174 | 0.149 | 0.046 | 0.079 | 0.088 | - | - | - | - | - | - | - |
| STCR | 0.146 | 0.134 | 0.154 | 0.149 | 0.162 | 0.149 | 0.053 | 0.066 | 0.085 | 0.030 | - | - | - | - | - | - |
| MISS-P15 | 0.167 | 0.145 | 0.161 | 0.163 | 0.178 | 0.165 | 0.059 | 0.085 | 0.107 | 0.010 | 0.047 | - | - | - | - | - |
| WPS | 0.144 | 0.132 | 0.143 | 0.150 | 0.158 | 0.142 | 0.041 | 0.053 | 0.072 | 0.007 | 0.033 | 0.005 | - | - | - | - |
| WIOR | 0.140 | 0.133 | 0.143 | 0.145 | 0.167 | 0.137 | 0.037 | 0.057 | 0.086 | 0.000 | 0.033 | 0.000 | 0.000 | - | - | - |
| WHIT-AR | 0.182 | 0.170 | 0.182 | 0.168 | 0.199 | 0.190 | 0.085 | 0.118 | 0.139 | 0.042 | 0.088 | 0.031 | 0.037 | 0.027 | - | - |
| WHIT-IN | 0.169 | 0.151 | 0.173 | 0.176 | 0.187 | 0.172 | 0.083 | 0.103 | 0.133 | 0.041 | 0.077 | 0.029 | 0.036 | 0.035 | 0.035 | - |
| WABASH | 0.187 | 0.170 | 0.191 | 0.186 | 0.202 | 0.180 | 0.097 | 0.112 | 0.141 | 0.047 | 0.087 | 0.047 | 0.051 | 0.040 | 0.042 | 0.003 |


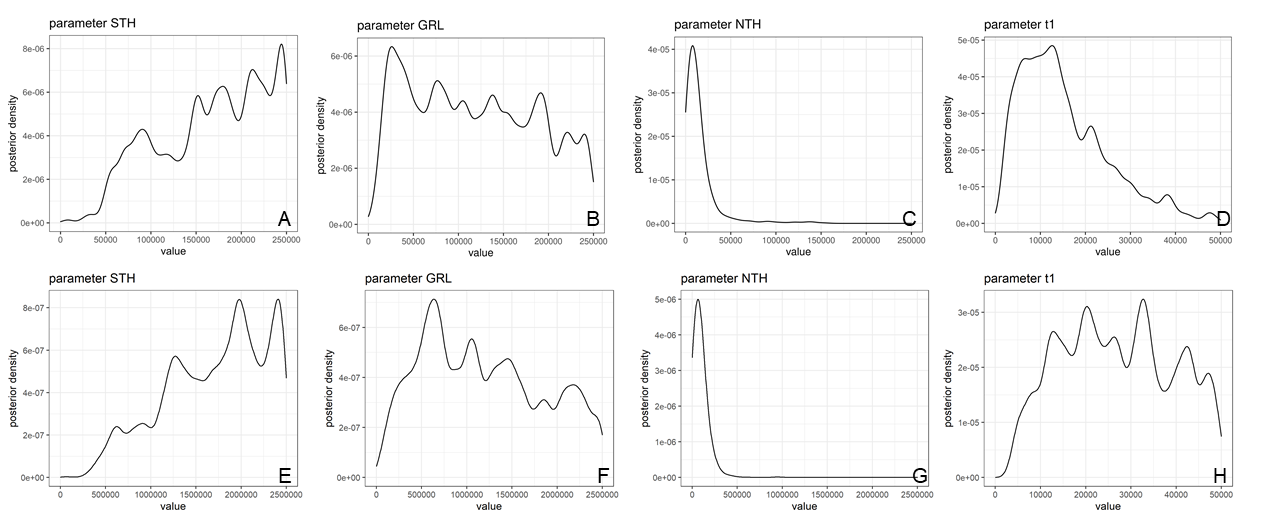


**Appendix S9)** DIYABC estimates for parameters based on model with 1) 250k N_e_ cap: A) Southern population N_e_ estimation B) Great Lakes population N_e_ estimation, C) Northern population N_e_ estimation, D) Time (in generations) estimation. 2) 2.5 million N_e_ cap: D) Southern populations N_e_ estimation, F) Great Lakes populations N_e_ estimation, G) Northern populations N_e_ estimation, H) Time (in generations) estimation.
